# Activation Studies of the γ-Carbonic Anhydrases from the Antarctic Marine Bacteria *Pseudoalteromonas haloplanktis* and *Colwellia psychrerythraea* with Amino Acids and Amines

**DOI:** 10.3390/md17040238

**Published:** 2019-04-22

**Authors:** Andrea Angeli, Sonia Del Prete, Sameh M. Osman, Zeid AlOthman, William A. Donald, Clemente Capasso, Claudiu T. Supuran

**Affiliations:** 1 Dipartimento Neurofarba, Sezione di Scienze Farmaceutiche e Nutraceutiche, Università degli Studi di Firenze, Via U. Schiff 6, Sesto Fiorentino, 50019 Florence, Italy; andrea.angeli@unifi.it; 2 Istituto di Bioscienze e Biorisorse, CNR, Via Pietro Castellino 111, 80131 Napoli, Italy; sonia.delprete@ibbr.cnr.it; 3 Department of Chemistry, College of Science, King Saud University, P.O. Box 2455 Riyadh 11451, Saudi Arabia; smahmoud@ksu.edu.sa (S.M.O.); zaothman@KSU.EDU.SA (Z.A.); 4 School of Chemistry, University of New South Wales, Dalton Building, Sydney, New South Wales 2052, Australia; w.donald@unsw.edu.au

**Keywords:** Antarctic bacteria, carbonic anhydrase, metalloenzymes, amine, amino acid, activator, *Pseudoalteromonas haloplanktis*, *Colwellia psychrerythraea*

## Abstract

The γ-carbonic anhydrases (CAs, EC 4.2.1.1) present in the Antarctic marine bacteria *Pseudoalteromonas haloplanktis* and *Colwellia psychrerythraea*, herein referred to as PhaCA and CpsCA, respectively, were investigated for their activation with a panel of 24 amino acids and amines. Both bacteria are considered Antarctic models for the investigation of photosynthetic and metabolic pathways in organisms adapted to live in cold seawater. PhaCA was much more sensitive to activation by these compounds compared to the genetically related enzyme CpsCA. The most effective PhaCA activators were d-Phe, l-/d-DOPA, l-Tyr and 2-pyridyl-methylamine, with the activation constant K_A_ values of 0.72–3.27 µM. d-His, l-Trp, d-Tyr, histamine, dopamine, serotonin anddicarboxylic amino acids were also effective activators of PhaCA, with K_A_ values of 6.48–9.85 µM. CpsCA was activated by d-Phe, d-DOPA, l-Trp, l-/d-Tyr, 4-amino-l-Phe, histamine, 2-pyridyl-methylamine and l-/d-Glu with K_A_ values of 11.2–24.4 µM. The most effective CpsCA activator was l-DOPA (K_A_ of 4.79 µM). Given that modulators of CAs from Antarctic bacteria have not been identified and investigated in detail for their metabolic roles to date, this research sheds some light on these poorly understood processes.

## 1. Introduction

*Pseudoalteromonas haloplanktis* and *Colwellia psychrerythraea* are bacteria adapted to live in the extreme conditions of the Antarctic Ocean. These marine heterotrophic bacteria are thought to rely on simplified metabolic strategies which evolved in order to overcome thermodynamic constraints connected toliving at low temperatures and in the dark for prolonged periods during the Antarctic winter [1,2,3,4,5,6,7]. Thus, these two bacteria are considered model organisms for studying metabolic processes and, more generally, strategies used for adaptation to harsh environmental conditions typical of the Antarctic region, which may also provide interesting insights for potential biotechnological or biomedical applications of enzymes encoded in their genomes [1,2,5].

Carbonic anhydrases (CAs, EC 4.2.1.1) are metalloproteins involved in several metabolic processes [8,9,10,11,12,13,14,15,16,17] either directly, by providing CO_2_/bicarbonate for carboxylating reactions [8,9,12,13], or indirectly, by modulating pH [13,14,15,16,17]. Indeed, CAs convert the neutral molecules CO_2_ and water to a weak base (bicarbonate) and a strong acid (hydronium ion) with very high efficacy [8,13]. For this reason, in many organisms including bacteria, CAs are the main players in pH homeostasis and related physiologic processes, which are involved in the metabolism, survival and colonization of various niches in which these organisms thrive [18,19,20,21,22,23,24,25]. These processes were mainly investigated for pathogenic microorganisms, such as bacteria [9,13,16,17], protozoa [10,11,21,22] and fungi [17,26]. However, the wide distributions of these enzymes in non-pathogenic Archaea [25] and Bacteria [3,4,6,7,27] make them of interest for understanding crucial biochemical processes connected with metabolism, adaptation to various environmental conditions, and survival in extreme environments. Indeed, we have reported CAs from extremophilic bacteria living at 110 °C in volcanic hot springs [28,29,30], as well as CAs from Antarctic organisms which thrive at very low temperatures, such as PhaCA from *P. haloplanktis* [3,4] and CpsCA from *C. psychrerythraea* [6,7]. Both these CAs belong to the γ-CA family, which is the least investigated CA family to date [9,12,13,25]. It should be mentioned that seven genetically unrelated CA families are known to date, the α-, β-, γ-, δ-, ζ-, η- and θ-CAs [12,23], and many of their inhibitors possess pharmacologic or environmental applications [14,15,31,32,33]. However, the activators of CA enzymes have been investigated in far less detail, with exception for the α-class enzymes. Indeed, activation of the human CAs, of which 15 isoforms are known to date [14,15], was recently shown to have the potential for pharmacologic applications in memory therapy and learning [34,35].

Here we report the first activation study of γ-CAs from Antarctic bacteria. We present an amino acid and amine activation study of PhaCA cloned from *P. haloplanktis*, and of CpsCA from *C. psychrerythraea*.

## 2. Results and Discussion

The catalytic activity of the recombinant γ-CAs from *P. haloplanktis* and *C. psychrerythraea* for the CO_2_ hydration reaction was reported earlier by our group [3,4,6,7]. Both enzymes showed a significant catalytic activity for the physiologic CO_2_ hydration reaction, with the following kinetic parameters: *k*_cat_ = 1.4 × 10^5^ s^−1^ and *k*_cat_/*K*_m_ = 1.9 × 10^6^ M^−1^·s^−1^ for PhaCA, and *k*_cat_ = 6.0 × 10^5^ s^−1^ and *k*_cat_/*K*_m_ = 4.7 × 10^6^ M^−1^·s^−1^ for CpsCA. Considering that these enzymes belong to the γ-CA class, which has the lowest CO_2_ hydrase activity of all CAs, the measured activities are indeed rather significant, especially when compared to those of other CAs (such as the human isoforms hCA I and II) used in our experiments as controls (Table 1). The activity of the enzymes PhaCA and CpsCA were also inhibited by acetazolamide (AZA, 5-acetamido-1,3,4-thiadiazole-2-sulfonamide), a clinically used sulfonamide inhibitor with K_I_ values ranging between 403 and 502 nM [3,4,6,7].

As for all CA classes investigated so far, the activators of the γ-CAs participate in the catalytic cycle [35,36,37,38,39,40,41,42], most probably by facilitating the rate-determining step of the catalytic cycle (shown schematically by the ping-pong Reactions (1) and (2) below). In fact, the rate-determining step for many CAs is the generation of the nucleophilic species of the enzyme (EZnOH), depicted by Equation (2):

(1) $${\mathrm{EZn}}^{2+}―{\mathrm{OH}}^{-}+{\mathrm{CO}}_{2}⇄{\mathrm{EZn}}^{2+}―{\mathrm{HCO}}_{3}^{-}\overset{H_{2}O}{⇄}{\mathrm{EZn}}^{2+}―{\mathrm{OH}}_{2}+{\mathrm{HCO}}_{3}^{-}$$

(2) $${\mathrm{EZn}}^{2+}―{\mathrm{OH}}_{2}⇄{\mathrm{EZn}}^{2+}―{\mathrm{OH}}^{-}+H^{+}$$

In all CAs, the step shown in Equation (2) is assisted by amino acid residues from the active site [35], which perform the function of shuttling protons between the catalytic active site and the solvent media. Thus, in the presence of sufficiently high concentrations of CA activators CAAs, the intermolecular proton transfer reaction (Equation (2)), becomes an intramolecular step within the enzyme–activator complex, which is favored thermodynamically. In this way, the activators (CAAs) participate in the rate-determining step of the catalytic cycle, which is represented by Equation (3):

(3) $$\begin{array}{c} {\mathrm{EZn}}^{2+}―{\mathrm{OH}}_{2}+A⇄[{\mathrm{EZn}}^{2+}―{\mathrm{OH}}_{2}–A]⇄[{\mathrm{EZn}}^{2+}―{\mathrm{OH}}^{-}–{\mathrm{AH}}^{+}]⇄{\mathrm{EZn}}^{2+}―{\mathrm{OH}}^{-}+{\mathrm{AH}}^{+}\\ \mathrm{enzyme}–\mathrm{activator}\;\mathrm{complexes} \end{array}$$

The enzyme–activator complexes (shown as E–A, where E stands for the enzyme and A for the activator) favor the intramolecular proton transfer step. In this way, the zinc-coordinated water loses a proton to the environment (in this intramolecular step), and promotes the formation of the catalytically active nucleophilic species of the enzyme, with the hydroxide ion coordinating the zinc ion [35]. In all X-ray crystal structures available so far for CAs complexed to activators, these molecules were observed to be bound within the active site crevice of the enzyme [35,36,37,38,39,40,41] in a region of the cavity that is distinct from the inhibitor binding site(s). However, to date, only the α-class CAs have been investigated in detail by this technique. Indeed, the activator binding sites for α-CAs such as hCA I and II (h = human isoform) were identified to be at the entrance of the active site cavity, near the amino acid residue His64 (hCA II numbering [35]), which is also the natural proton shuttle residue for enzymes belonging to the α-class [35,36,37,38,39,40,41]. It should be mentioned that the nature of the amino acid residue(s) involved in the proton shuttling for the γ-CAs is for the moment rather controversial, although several proposals were reported, such as for example two Glu residues (for CAM, the first such enzyme investigated in detail by Ferry’s group from the archaeon *Methanosarcina thermophila* [25]) situated at the entrance of the cavity.

We measured the kinetic constants (k_cat_ and K_M_) of the two γ-CAs (for the physiologic CO_2_ hydration reaction) investigated here in the presence of 10 μM l-Trp as an activator in order to see whether the activation mechanism is different from that of the α-class enzymes investigated earlier [35]. The data of Table 1 show that the presence of l-Trp does not change the value of K_M_ for both of the two enzymes belonging to the α-class (hCA I/II) as well as for the two bacterial Antarctic enzymes PhaCA and CpsCA. In fact, the activator has an effect only on the k_cat_, which at a 10 µM concentration of the activator leads to an enhancement of 5.42 times of the kinetic constant for PhaCA, and of 1.65 times for CpsCA (Table 1). The same type of enhancement of the k_cat_ with no influence on K_m_ was observed for hCA I and II [35], indicating that the activation mechanisms of the two enzyme classes must be rather similar.

**Figure 1:**
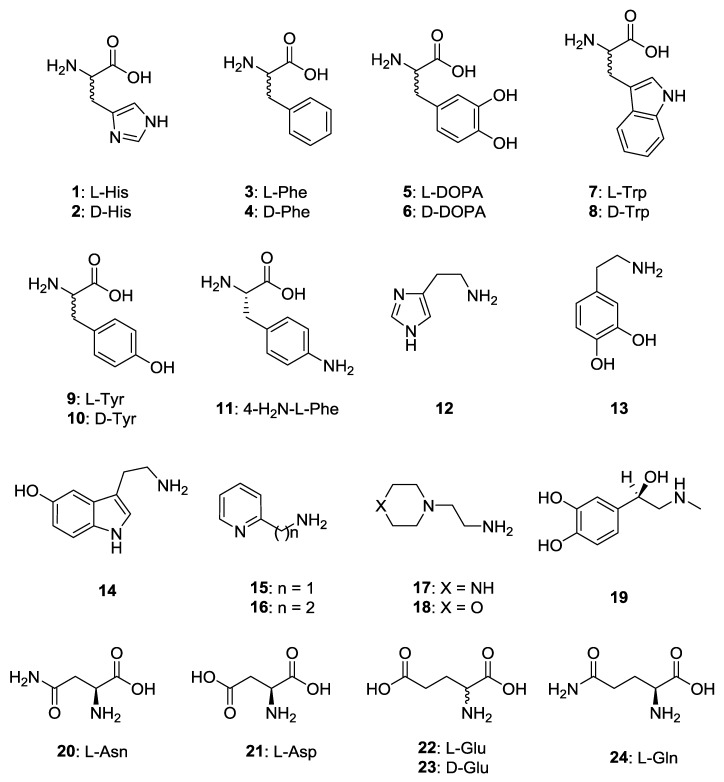
CA activators CAAs of types **1**–**24** used in the present study.

We report the activation profiles of PhaCA and CpsCA with a wide range of amino acid and amine activators of types **1**–**24** (Figure 1), which were obtained by measuring the dose response curves of the activation of the two enzymes in the presence of increasing concentrations of activators. It should be mentioned that in order to act as CAAs, the amino and COOH moiety of the inhibitor should generally not be derivatized [35]. Small modifications such as N-methylation or the transformation of the COOH to COOMe groups are tolerated and do not significantly change the CA activating properties, as demonstrated earlier by us [35,40,41]. This is the reason why we include amines and amino acids **1**–**24** in our study and not some of their derivatives. In this way, the K_A_ values of the 24 compounds were determined for the two enzymes (see Materials and Methods for details). We included in our study the amino acids and amines that were investigated as activators of CAs belonging to various classes of enzymes present in diverse organisms, both eukaryotic and prokaryotic [32,33,34,35,36,37,40,41,42].

The structure–activity relationship (SAR) for the activation of PhaCA/CpsCA with compounds **1**–**24** revealed the following observations (Table 2):

(i) Firstly, PhaCA is much more sensitive to activation with amines and amino acids **1**–**24**, compared to CpsCA, although both enzymes are prokaryotic, belonging to the same class. Indeed, the activation constants of these amines ranged between 0.72 and 32.4 µM for PhaCA, whereas they were only in the range of 4.79 to 100 µM for CpsCA.
(ii) The most effective PhaCA activators were d-Phe, l-/d-DOPA, l-Tyr and amine **15**, which showed K_A_ values ranging between 0.72 and 3.27 µM. Compounds such as d-His, l-Trp, d-Tyr, histamine, dopamine, serotonin and **20**–**23** were also effective activators, with K_A_ values ranging between 6.48 and 9.85 µM. Medium potency activators were l-His, l-Phe, d-Trp, amines **16**–**19** and l-Gln, which showed K_A_ values ranging between 10.1 and 32.4 µM (Table 1). Thus, the SAR was rather complex, but generally the d-amino acids were better activators than their l-enantiomers (except for the enantiomer pairs l-/d-Trp and l-/d-Tyr). In some cases, the amines (histamine) were more effective activators than the structurally related amino acids (l- and d-His), whereas in other cases (l-/d-DOPA) the amino acids were more effective CAAs compared to the structurally related amine (dopamine). No major differences were observed between carboxylate/carboxamide derivatives in some cases (l-Asp/l-Asn), whereas for l-Glu, the carboxamide (l-Gln) was more than three times less effective as an activator.
(iii) For CpsCA, l-Phe and l-Gln were devoid of activating effects up to a 100 µM concentration of compound in the assay system. Weak activators were also l-/d-His, d-Trp, dopamine, serotonin, amines **16**–**19**, as well as l-Asn and l-Asp (K_A_ values ranging between 27.3 and 79.8 µM). On the other hand, better activators of CpsCA were d-Phe, d-DOPA, l-Trp, l-/d-Tyr, 4-amino-l-Phe, histamine, 2-pyridyl-methylamine and l-/d-Glu, with K_A_ values ranging between 11.2 and 24.4 µM. The most effective CpsCA activator was l-DOPA, with a K_A_ of 4.79 µM. It is obvious that very small structural changes in the molecule of the activator have drastic effects on the enzyme activating effects. For example, the two enantiomers of DOPA have values of K_A_ which differ by a factor of 2.33, with the l-enantiomer being the most active. However, the amino acid in which one of the two OH moieties of DOPA is missing, l-Tyr, was 4 times less effective compared to DOPA, whereas l-Phe was more than 20 times less effective as an activator of this isoform (the compound lacking both phenolic OH groups present in DOPA).
(iv) As no X-ray crystal structure for adducts of activators with γ-CAs are available so far, we cannot rationalize our SAR data in detail. However, all the observations reported above concur with the fact that these compounds bind within the enzyme active site and facilitate the generation of the nucleophilic zinc hydroxide species of the enzyme.

## 3. Materials and Methods

The protocol described in [3,4,6,7] was used to obtain purified recombinant *Pseudoalteromonas haloplanktis* (PhaCA) and *Colwellia psychrerythraea* (CpsCA) enzymes.

### CA Activity/Activation Measurements

An Sx.18Mv-R Applied Photophysics (Oxford, UK) stopped-flow instrument was used to assay the catalytic activity of various CA isozymes for CO_2_ hydration reaction [42]. Phenol red (at a concentration of 0.2 mM) was used as an indicator, working at the absorbance maximum of 557 nm, with 10 mM Hepes (pH 7.5, for α-CAs) or TRIS (pH 8.3, for γ-CAs) as buffers, and 0.1 M NaClO_4_ (for maintaining constant ionic strength), following the CA-catalyzed CO_2_ hydration reaction for a period of 10 s at 25 °C. The CO_2_ concentrations ranged from 1.7 to 17 mM for the determination of the kinetic parameters and inhibition constants. For each activator, at least six traces of the initial 5–10% of the reaction were used to determine the initial velocity. The uncatalyzed rates were determined in the same manner and subtracted from the total observed rates. Stock solutions of activators (at 0.1 mM) were prepared in distilled-deionized water and dilutions up to 1 nM were made thereafter with the assay buffer. Enzyme and activator solutions were pre-incubated together for 15 min prior to the assay, in order to allow for the formation of the enzyme–activator complexes. The activation constant (K_A_), defined similarly as the inhibition constant K_I_, can be obtained by considering the classical Michaelis–Menten equation (Equation (4)), which was fitted by non-linear least squares by using PRISM 3:

(4) $$v=v_{\max}/\{1+(K_{M}/[S])(1+{\left[A\right]}_{f}/K_{A})\}$$

where [A]_f_ is the free concentration of activator.

Working at substrate concentrations considerably lower than K_M_ ([S] << K_M_), and considering that [A]_f_ can be represented in the form of the total concentration of the enzyme ([E]_t_) and activator ([A]_t_), the obtained competitive steady-state equation for determining the activationconstant is given by Equation (5):

(5) $$v=v_{0}\times K_{A}/\{K_{A}+({\left[A\right]}_{t}-0.5{\left\{({\left[A\right]}_{t}+{\left[E\right]}_{t}+K_{A})-{\left({\left[A\right]}_{t}+{\left[E\right]}_{t}+K_{A}\right)}^{2}-4{\left[A\right]}_{t}\times {\left[E\right]}_{t}\right)}^{1/2}\}\}$$

where *v*_0_ represents the initial velocity of the enzyme-catalyzed reaction in the absence of an activator [43,44,45]. The equilibrium constants measured using this approach are in excellent agreement with those obtained from other methods, including native mass spectrometry and fluorescence spectroscopy [46].

## 4. Conclusions

γ-CAs are present in bacteria, archaea and plants, constituting probably the most archaic class of such enzymes [9]. In the Antarctic marine bacteria *Pseudoalteromonas haloplanktis* and *Colwellia psychrerythraea*, two such enzymes—PhaCA and CpsCA, respectively—were recently reported. However, their role in the life cycle of these prokaryotes is poorly investigated. However, both these bacteria are considered model Antarctic organisms for the investigation of photosynthetic and metabolic pathways in organisms adapted to live in extreme conditions that are characterized by cold seawater and absence of light during the Antarctic winter. A panel of 24 amino acid and amines were investigated as activators of the two enzymes. PhaCA was much more sensitive to activation by these compounds compared to the genetically related enzyme CpsCA. The best PhaCA activators were d-Phe, l-/d-DOPA, l-Tyr and 2-pyridyl-methylamine, with K_A_ values of 0.72–3.27 µM. d-His, l-Trp, d-Tyr, histamine, dopamine, serotonin and dicarboxylic amino acids were also effective activators, with K_A_ values of 6.48–9.85 µM. CpsCA was activated by d-Phe, d-DOPA, l-Trp, l-/d-Tyr, 4-amino-l-Phe, histamine, 2-pyridyl-methylamine and l-/d-Glu, with K_A_ values of 11.2–24.4 µM. The most effective CpsCA activator was l-DOPA (K_A_ of 4.79 µM). Sincemodulators of CAs from Antarctic bacteria have not yet been investigated in detail for their metabolic roles, these data provide valuable insights into these understudied processes.

## Figures and Tables

**Table 1:** Activation of human carbonic anhydrase (hCA) isozymes I, II, PhaCA and CpsCA with l-Trp, at 25 °C, for the CO_2_ hydration reaction [42].

Isozyme	k_cat_^1^ (s^−1^)	K_M_^1^ (mM)	(k_cat_) l-Trp ^2^ (s^−1^)	K_A_^3^ (μM) l-Trp
hCA I ^a^	2.0 × 10^5^	4.0	3.4 × 10^5^	44.0
hCA II ^a^	1.4 × 10^6^	9.3	4.9 × 10^6^	27.0
PhaCA ^b^	1.4 × 10^5^	7.3	7.6 × 10^5^	7.12
CpsCA ^b^	6.0 × 10^5^	12.7	9.9 × 10^5^	21.3

^1^ Observed catalytic rate without activator. K_M_ values in the presence and the absence of activators were the same for the various carbonic anhydrases (CAs) (data not shown). ^2^ Observed catalytic rate in the presence of 10 μM activator. ^3^ The activation constant (K_A_) for each enzyme was obtained by fitting the observed catalytic enhancements as a function of the activator concentration [41]. The mean was obtained from at least three determinations by a stopped-flow CO_2_ hydrase method [42]. Standard errors were in the range of 5–10% of the reported values (data not shown). ^a^ Human recombinant isozymes, from [32]; ^b^ Antarctic bacteria recombinant enzyme, this work.

**Table 2:** Activation constants of hCA I, hCA II and the bacterial enzymes PhaCA (*Pseudoalteromonas haloplanktis*) and CpsCA (*Colwellia psychrerythraea*) with amino acids and amines **1**–**24**, by a stopped-flow CO_2_ hydrase assay [42].

No.	Compound	K_A_ (μM) *			
		hCA I ^a^	hCA II ^a^	PhaCA ^b^	CpsCA ^b^
**1**	l-His	0.03	10.9	12.6	47.5
**2**	d-His	0.09	43	9.41	35.9
**3**	l-Phe	0.07	0.013	15.8	>100
**4**	d-Phe	86	0.035	3.19	15.4
**5**	l-DOPA	3.1	11.4	1.08	4.79
**6**	d-DOPA	4.9	7.8	0.72	11.2
**7**	l-Trp	44	27	7.12	21.3
**8**	d-Trp	41	12	13.9	36.8
**9**	l-Tyr	0.02	0.011	1.02	19.5
**10**	d-Tyr	0.04	0.013	7.35	18.4
**11**	4-H_2_N-l-Phe	0.24	0.15	3.27	17.2
**12**	Histamine	2.1	125	6.48	20.6
**13**	Dopamine	13.5	9.2	8.70	32.1
**14**	Serotonin	45	50	9.05	34.8
**15**	2-Pyridyl-methylamine	26	34	2.39	21.5
**16**	2-(2-Aminoethyl)pyridine	13	15	18.7	38.2
**17**	1-(2-Aminoethyl)-piperazine	7.4	2.3	15.1	33.0
**18**	4-(2-Aminoethyl)-morpholine	0.14	0.19	10.1	34.3
**19**	l-Adrenaline	0.09	96.0	17.5	79.8
**20**	l-Asn	11.3	>100	9.80	27.9
**21**	l-Asp	5.20	>100	9.85	27.3
**22**	l-Glu	6.43	>100	9.01	24.4
**23**	d-Glu	10.7	>100	4.72	12.0
**24**	l-Gln	>100	>50	32.4	>100

* Means were obtained from three determinations by a stopped-flow CO_2_ hydrase method [42]. Standard errors were in the range of 5–10% of the reported values (data not shown). ^a^ Human recombinant isozymes, from [35]; ^b^ Antarctic bacterial recombinant enzyme, this work.
